# Temporal patterns of cytokine and injury biomarkers in hospitalized COVID-19 patients treated with methylprednisolone

**DOI:** 10.3389/fimmu.2023.1229611

**Published:** 2023-08-16

**Authors:** Victor Irungu Mwangi, Rebeca Linhares Abreu Netto, Carlos Eduardo Padron de Morais, Arineia Soares Silva, Bernardo Maia Silva, Amanda Barros Lima, Juliana Costa Ferreira Neves, Mayla Gabriela Silva Borba, Fernando Fonseca de Almeida e Val, Anne Cristine Gomes de Almeida, Allyson Guimarães Costa, Vanderson de Souza Sampaio, Luiz Gustavo Gardinassi, Marcus Vinicius Guimarães de Lacerda, Wuelton Marcelo Monteiro, Gisely Cardoso de Melo

**Affiliations:** ^1^ Programa de Pós-Graduação em Medicina Tropical, Universidade do Estado do Amazonas (UEA), Manaus, Brazil; ^2^ Fundação de Medicina Tropical Heitor Vieira Dourado (FMT-HVD), Manaus, Brazil; ^3^ Programa de Pós-Graduação em Imunologia Básica e Aplicada, Instituto de Ciências Biológicas, Universidade Federal do Amazonas (UFAM), Manaus, Brazil; ^4^ Diretoria de Ensino e Pesquisa, Fundação Hospitalar de Hematologia e Hemoterapia do Amazonas (HEMOAM), Manaus, Brazil; ^5^ Instituto de Patologia Tropical e Saúde Pública, Universidade Federal de Goiás (UFG), Goiânia, Brazil; ^6^ Escola de Enfermagem de Manaus, Universidade Federal do Amazonas (UFAM), Manaus, Brazil; ^7^ Programa de Pós-Graduação em Ciências Aplicadas à Hematologia, Fundação Hospitalar de Hematologia e Hemoterapia do Amazonas (HEMOAM) Universidade do Estado do Amazonas (UEA), Manaus, Brazil; ^8^ Instituto Todos pela Saúde, São Paulo, São Paulo, Brazil; ^9^ Instituto Leônidas & Maria Deane/Fundação Oswaldo Cruz (ILMD/Fiocruz Amazônia), Manaus, Brazil

**Keywords:** SARS-CoV-2, methylprednisolone, biomarkers, injury, cytokine, immune mediators

## Abstract

**Background:**

The novel coronavirus disease 2019 (COVID-19) presents with complex pathophysiological effects in various organ systems. Following the COVID-19, there are shifts in biomarker and cytokine equilibrium associated with altered physiological processes arising from viral damage or aggressive immunological response. We hypothesized that high daily dose methylprednisolone improved the injury biomarkers and serum cytokine profiles in COVID-19 patients.

**Methods:**

Injury biomarker and cytokine analysis was performed on 50 SARS-Cov-2 negative controls and 101 hospitalized severe COVID-19 patients: 49 methylprednisolone-treated (MP group) and 52 placebo-treated serum samples. Samples from the treated groups collected on days D1 (pre-treatment) all the groups, D7 (2 days after ending therapy) and D14 were analyzed. Luminex assay quantified the biomarkers HMGB1, FABP3, myoglobin, troponin I and NTproBNP. Immune mediators (CXCL8, CCL2, CXCL9, CXCL10, TNF, IFN-γ, IL-17A, IL-12p70, IL-10, IL-6, IL-4, IL-2, and IL-1β) were quantified using cytometric bead array.

**Results:**

At pretreatment, the two treatment groups were comparable demographically. At pre-treatment (D1), injury biomarkers (HMGB1, TnI, myoglobin and FABP3) were distinctly elevated. At D7, HMGB1 was significantly higher in the MP group (p=0.0448) compared to the placebo group, while HMGB1 in the placebo group diminished significantly by D14 (p=0.0115). Compared to healthy control samples, several immune mediators (IL-17A, IL-6, IL-10, MIG, MCP-1, and IP-10) were considerably elevated at baseline (all p≤0.05). At D7, MIG and IP-10 of the MP-group were significantly lower than in the placebo-group (p=0.0431, p=0.0069, respectively). Longitudinally, IL-2 (MP-group) and IL-17A (placebo-group) had increased significantly by D14. In placebo group, IL-2 and IL-17A continuously increased, as IL-12p70, IL-10 and IP-10 steadily decreased during follow-up. The MP treated group had IL-2, IFN-γ, IL-17A and IL-12p70 progressively increase while IL-1β and IL-10 gradually decreased towards D14. Moderate to strong positive correlations between chemokines and cytokines were observed on D7 and D14.

**Conclusion:**

These findings suggest MP treatment could ameliorate levels of myoglobin and FABP3, but appeared to have no impact on HMGB1, TnI and NTproBNP. In addition, methylprednisolone relieves the COVID-19 induced inflammatory response by diminishing MIG and IP-10 levels. Overall, corticosteroid (methylprednisolone) use in COVID-19 management influences the immunological molecule and injury biomarker profile in COVID-19 patients.

## 1 Introduction

The ongoing Coronavirus disease 2019 (COVID-19) pandemic has presented a major challenge to clinicians, medical scientists and patients as well as existing healthcare systems. This very contagious respiratory infection is associated with a large spectrum of symptoms, a myriad of sequelae, complications, and death.

The etiological agent of the COVID-19, that is the severe acute respiratory syndrome coronavirus 2 (SARS-CoV-2), attaches and enters target human cells via the human host’s angiotensin converting enzyme receptor 2 (ACE2) and neuropilin-1 (NRP1) cell receptors with the activation of the serine transmembrane protease 2 (TMPRSS2) host receptor as it enters the target cells. The target cells with these receptors are mostly found in the respiratory and gastrointestinal tract receptors (1, 2). Subsequently, the viral invasion causes a complex interplay of the immunological, inflammatory, and coagulative cascades (3–7). In addition to the organ tropism demonstrated by the virus, SARS-CoV-2 infection can influence the course of the disease by potentially aggravating other underlying conditions, of which the systemic responses to the viral infection may cause organ dysfunction in affected systems (3–5).

In majority of the cases, the clinical features of COVID-19 presented with acute respiratory distress syndrome (ARDS) that could quickly evolve into pneumonia, respiratory failure, and death. The ARDS is often accompanied by a rise in the levels of inflammatory cytokines such as IL-2, IL-7, IL-6, IL-10, IP-10, MCP1 and TNF-α (8, 9), referred to as the cytokine storm. At the same time elevated serum and plasma levels of cytokines like IL-6, IL-10, TNF-α and IL-12R have been associated with disease severity (1, 10). To attenuate the cytokine storm effects and manage the cytokine-induced injury, corticosteroids such as methylprednisolone have been administered as therapeutic intervention to COVID-19 patients (11–18). Borrowing from the methylprednisolone use in treating Severe Acute Respiratory Syndrome Coronavirus (SARS-CoV) and Middle East respiratory syndrome coronavirus (MERS-CoV) outbreaks (19–22), corticosteroids became one of the first-line therapeutic intervention against COVID-19.

Understanding the host-pathogen interaction in COVID-19 is vital for improved treatment and management of the disease. With this, biomarkers to disease progression, prognosis, and response to treatment are important. Studies on COVID-19 patients demonstrated changes in damage associated molecular patterns (DAMPs) including high mobility group box 1 (HMGB1) and surfactant protein A (SP-A) (23–25). Other biomarkers like myoglobin and troponin I have been positively correlated with significantly higher risks of severe disease and mortality among COVID-19 patients (26–29). Data from meta-analyses further highlight the prognostic value of the cardiac markers, both Brain natriuretic peptides (BNP), and N-terminal pro-B-type natriuretic peptide (NT-proBNP) with regard to mortality and disease severity in patients with COVID-19 (28, 30–34). Similarly, COVID-19 has been associated with increased levels neuronal injury serum markers like neurofilament light chain (NfL), neuron-specific enolase (35–37) while biomarkers like NfL, Glial fibrillary acidic protein, and tau were significantly increased in patients with fatal outcome (38). From these studies, elevated levels of these biomarkers, among others, are often indicative of unfavorable outcomes and other complications in COVID-19.

Some works report different biomarkers that related to the state of the affected organs and systems, information about the progression/improvement of the disease and the response to treatment (3, 39–44). Immunological, inflammatory, coagulation, hematological, cardiac, biochemical, damage-associated molecular patterns and abnormal laboratory markers are some of the informative biomarkers used in COVID-19 investigations (3). Understanding the profiles of biomarkers and cytokines in infection, as well as the changes that occur after administration of systemic corticosteroid treatment, is critical to assessing the benefits and/or risks of this treatment (3).

Monitoring levels of immunological and tissue injury biomarkers is important to understand the dynamics of corticosteroid therapeutic responses in patients with COVID-19, as well as their effects. These biomolecules may serve as prognostic factors and/or biomarkers of therapeutic success in patients with COVID-19. This work aimed to determine the effect of methylprednisolone on tissue injury biomarkers and cytokine profiles compared to placebo in patients with COVID-19.

## 2 Methods

This was an analytical study using samples and secondary data from retrospective cohort, patients from the MetCOVID clinical trial (45, 46). In our study, baseline serum cytokine and injury biomarker profiles of COVID-19 infected patients who completed the 5-day methylprednisolone (MP) treatment and, the longitudinal effect of the MP intervention at 14-day follow-up on the cytokine and biomarker changes was determined and compared to the placebo group. Patient demographic and clinical data were also targeted for analysis.

### 2.1 Ethics statement

The *Fundação de Medicina Tropical Doutor Heitor Vieira Dourado* Research Ethics Committee approved this study under certificate CAAE 46193821.5.0000.0005. This was a sub study of a larger clinical trial study approved under certificate number 30615920.2.0000.0005. Likewise, the *Fundação Hospitalar de Hematologia e Hemoterapia do Amazonas* (HEMOAM) Ethics Committee approved the negative control group (CAAE 56413316.9.0000.0009). All donors were submitted to a serological screening at HEMOAM, which is recommended by Brazilian Blood Donor Bank Authorities in order to monitor blood borne pathogens and includes serological analysis for the Hepatitis B and C virus, HIV, DENV, HTLV, Syphilis and Chagas Disease.

### 2.2 The participants

The participants used in this study were drawn from the MetCOVID study (Clinical Trials Identifier NCT04343729). The MetCOVID clinical trial was a double-blinded, randomized, placebo-controlled, phase IIb clinical trial that aimed to assess the efficacy of intravenous methylprednisolone sodium succinate (MP), compared to placebo treatment. The methylprednisolone sodium succinate treatment (0.5 mg/kg) was administered twice daily for 5 days in hospitalized patients with suspected COVID-19 infection, while the placebo arm of the study received saline solution twice daily for 5 days (45, 46).

For our study, we selected participants of either gender that had no prior use of corticosteroids before enrollment into the MetCOVID trial and had completed their 5-day treatment dose, in both the methylprednisolone and placebo groups. An additional negative control group (n=50) was included for comparison in the cytokine analysis. These control samples, collected only time, were collected from donors at the *Fundação Hospitalar de Hematologia e Hemoterapia do Amazonas* (HEMOAM), the principal hematological reference hospital in Manaus city, Amazonas, Brazil, prior to the outbreak of the COVID-19 pandemic. The control samples were thus COVID-19 negative and were analyzed only during the baseline (D1) comparison with the two treatment groups.

Subsequent information on patient demographics, clinical laboratory analyses (hematological and biochemical) were extracted from the REDCap electronic database. Hematological and biochemical tests at baseline were conducted as described by Jeronimo et al. (45).

### 2.3 Biological sample collection and processing

Venous blood was collected into vacutainer tubes containing clot activator on days D1 (pre-treatment), D7, and D14. Serum was collected and kept at -80°C until needed.

### 2.4 Quantification of biomarkers

Serum levels of NTproBNP, Troponin I, Myoglobin, FABP3, and HMGB1 were determined using commercially available bead-based immunoassay kits (MILLIPLEX MAP HCVD1MAG-67K, HCVD2MAG-67K, HNS2MAG-95K, and HCYP4MAG-64K kits; Merck KGaA). The assay was performed following the manufacturer’s instructions. Serum dilution was adapted to 1:4, 1:10 and 1:100 for HMGB1, FABP3 and myoglobin, respectively, to improve the assay range of target analytes. Samples, standards, and controls were run in duplicate with a coefficient of variance (CV) <20%. For readout and data acquisition, a MAGPIX® reader (Luminex Corporation) with xPONENT® software (version 4.3) was used, while Curve fitting was done using Belysa® Curve Fitting Software version 1.0.19 (Merck KGaA, Darmstadt, Germany).

### 2.5 Quantification of chemokine and cytokines

Soluble serum immunological mediators (chemokine and cytokine) including CXCL8, CCL2, CXCL9, CXCL10, TNF, IFN-γ, IL-17A, IL-12p70, IL-10, IL-6, IL-4, IL-2 and IL-1β were quantified using arrays of cytometric beads (Human Chemokine, Human Inflammatory Cytokine and Human Cytokine Th1/Th2/Th17 BD™ kits, BD Biosciences, San Diego, CA, USA), according to the manufacturer’s instructions. Data was acquired using the FACSCanto II and analyzed using the FCAP-Array software version 3.0.1 (BD Biosciences, San Jose, CA, USA). Data were reported in picograms per milliliter (pg/mL) concentrations, according to the standard curves provided in the kits.

### 2.6 Statistical analysis

Descriptive statistics was done for demographic, laboratory and clinical data. For qualitative variables, the chi-square test was performed. Statistical significance in the differences in quantitative variables was assessed using parametric (unpaired t-test) or non-parametric (unpaired Mann-Whitney test) tests, after initially being subjected to a Shapiro-Wilk test for normality. ANOVA test (normally distributed variables) and two-tailed Kruskal-Wallis test with Dunn’s post-test (non-parametric variables) were used in multi-group comparisons. These tests were also done for biomarker and cytokine assay data. Cytokine results were log-transformed before doing the comparative statistical analysis. In all cases, significance was considered at *p* < 0.05. Statistical analyses were performed using STATA version 15.0 and GraphPad Prism, version 9.0.1 (GraphPad Software, San Diego, CA, USA).

### 2.7 Correlation network analysis

Cytokine correlation networks were set up to assess associations in the MP and placebo groups on D1, D7, and D14.Correlations between quantitative levels of chemokines and cytokines were determined using Spearman’s correlation coefficient in GraphPad Prism, version 9.0.1 (GraphPad Software, San Diego, CA, USA). After performing a correlation matrix analysis between immunological molecules, a database was created using the Microsoft Excel program, where only significant correlations were considered for further network development using the open source Cytoscape software (version 3.9.1) (Cytoscape Consortium San Diego, CA, USA). Following the software’s recommendations and instructions, the chemokine and cytokine networks were constructed. The correlation index (r) was used to categorize the correlation strength as weak (r ≤ 0.35), moderate (r ≥ 0.36 to r ≤ 0.67) or strong (r ≥ 0.68), as proposed earlier (47, 48). The levels of statistical significance defined in both cases were p < 0.05.

## 3 Results

### 3.1 Characterizing the baseline clinical, epidemiological and laboratory data of the patients

Our study included 101 patients categorized into two groups, 49 from MP group and 52 from the Placebo group. The patients had a median age of 60 years, were overweight (BMI=28 kg/m^2^), mostly males (68.3%) and mostly of an admixed racial background (79.2%). Most participants (91%) reported having suffered other comorbidities, particularly hypertension (59.8%), obesity (37.0%) and diabetes (29.4%) respectively. Most of the participants having a Glasgow coma score of between 13 and 15, the patients had little to mild brain injuries. The laboratory findings of neutrophils, platelets, blood glucose, ALT, AST, D-dimer, and IL-6 were observed to be above the reference values. On the other hand, lymphocyte levels were below the reference values. Overall, there were no significant differences in the baseline characteristics between patients randomized into the MP and placebo groups (**Table 1**).

**Table 1:** Baseline demographic, clinical and laboratory results at randomization.

Characteristics	Total (n=101)	MP (n=49)	Placebo (n=52)	p-value
Age, years, median(IQR)	60 (49-69)	60 (46-69)	60 (50.5-72)	0.646
BMI, kg/m^2^, median(IQR)	28 (25.4 - 31.0)	27.8 (24.6 - 31.1)	28.4 (25.8 - 31.0)	0.409
Sex				
Female, n/N (%)	32 (31.7)	15 (30.6)	17 (32.7)	0.822
Male, n/N (%)	69 (68.3)	34 (69.4)	35 (67.3)	
Race, n/N (%)				
White	9 (8.9)	3 (6.1)	6 (11.5)	0.195
Mixed race (Brown)	80 (79.2)	43 (87.8)	37 (71.2)	
Black	6 (5.9)	1 (2.0)	5 (9.6)	
Amerindian/Indigenous	6 (5.9)	2 (4.1)	4 (7.7)	
Days from illness onset to randomization, median (IQR)	11.5 (8 - 15)	12 (8 - 15)	11 (8 - 14)	0.416
ICU on admission, n/N (%)	12 (11.9)	5 (10.2)	7 (13.5)	0.613
Intubated, n/N (%)	12 (11.9)	5 (10.2)	7 (13.5)	0.613
Glasgow Coma Scale Score				
3	12 (11.9)	4 (8.2)	8 (15.4)	
13	3 (3.0)	2 (4.1)	1 (1.9)	0.556
14	6 (5.9)	4 (8.2	2 (3.85)	
15	80 (79.2)	39 (79.6)	41 (78.9)	
Pre-existing morbidities, n/N (%)	92/101 (91.1)	46/49 (93.9)	46/52 (88.5)	0.340
Hypertension	55/92 (59.8)	26/46 (56.52)	29/46 (63.0)	0.524
Obesity	34/92 (37.0)	16/46 (34.8)	18/46 (39.1)	0.666
Diabetes	27/92 (29.4)	11/46 (23.9)	16/46 (34.8)	0.252
Alcohol Use	19/92 (20.7)	12/46 (26.1)	7/46 (15.2)	0.344
Liver disease	10/92 (10.9)	2/46 (4.4)	8/46 (17.4)	0.074
Smoking (currently)	4/92 (4.4)	3/46 (6.5)	1/46 (2.2)	0.753
Coronary heart disease	9/92 (9.8)	4/46 (8.9)	5/46 (10.9)	0.726
Chronic Respiratory Disease	4/92 (4.4)	2/46 (4.4)	2/46 (4.4)	>0.999
Previous TB	3/92 (3.3)	2/46 (4.4)	1/46 (2.2)	0.502
TB in treatment	1/92 (1.1)	1/46 (2.2)	0/46 (0)	>0.999
Chronic hematological disease	2/92 (2.2)	2/46 (4.4)	0/46 (0)	0.495
Neurological disease	2/92 (2.2)	1/46 (2.2)	1/46 (2.2)	>0.999
Medication Use, n/N (%)				
Antibiotics	76/101 (75.3)	38/49 (77.6)	38/52 (73.1)	0.603
Azithromycin	57/73 (78.1)	27/37 (73.0)	30/36 (83.3)	0.285
Bronchodilators	10/101 (9.9)	4/49 (8.2)	6/52 (11.5)	0.570
Statins	5/101 (5.0)	2/49 (4.1)	3/52 (5.8)	1.000
Calcium blockers	3/101 (3.0)	3/49 (6.1)	0/52 (0)	0.111
ACE inhibitors	48/101 (47.5)	19/49 (38.8)	29/52 (55.8)	0.087
Other remedies	99/101 (98.0)	47/49 (95.9)	52/52 (100)	0.141
Vital signs				
SpO2 on admission, median (IQR)	96 (94 - 98)	96 (94 - 98)	96 (94 - 98)	0.535
Pulse beats per minute, median (IQR)	86 (79-95)	86 (79 - 94)	88 (76.5 - 98.5)	0.940
Systolic blood pressure, mmHg, mean (SD)	132.4 (20.4)	130.1 (20.3)	134.6 (20.5)	0.273
Blood Pressure, mmHg, mean (SD)	98.3 (15.2)	97.8 (15.6)	98.7 (14.9)	0.751
Temperature, median (IQR)	36.2 (35.8 - 36.7)	36.3 (35.9 - 36.7)	36.1 (35.8 - 36.6)	0.158
Symptoms				
Fatigue	94/101 (93.1)	44/49 (89.8)	50/52 (96.2)	0.209
Cough	89/101 (88.1)	42/49 (85.7)	47/52 (90.4)	0.468
Fever	88/101 (87.1)	43/49 (87.8)	45/52 (86.5)	0.855
Breathlessness	88/101 (87.1)	42/49 (85.7)	46/52 (88.5)	0.680
Headaches	76/101 (75.3)	42/49 (85.7)	34/52 (65.4)	0.018
Myalgia	72/101 (71.3)	35/49 (71.4)	37/52 (71.2)	0.574
Diarrhea	65/101 (64.4)	34/49 (69.4)	31/52 (59.6)	0.305
Sputum	62/101 (61.4)	30/49 (61.2)	32/52 (61.5)	0.974
Nausea	58/100 (58.0)	29/48 (60.4)	29/52 (55.8)	0.638
Vomiting	35/101 (34.7)	17/49 (34.7)	18/52 (34.6)	0.993
Nausea	58/100 (58.0)	29/48 (60.4)	29/52 (55.8)	0.638
qSOFA score ≥ 2, n/N(%)	18/101 (17.8)	10/49 (20.4)	8/52 (15.4)	0.510
Laboratory findings				
White blood cell count,×10^3^/mm^3^, median (IQR)	9.6 (7.7 - 13.3)	9.5 (8.0 - 14.8)	9.6 (7.1 - 13.0)	0.464
Neutrophils, %, median (IQR) ↑	82.4 (72.6 -88.4)	82.2 (75.4 - 89.2)	82.5 (72.1 - 8.74)	0.242
Lymphocytes, %, median (IQR) ↓	11.3 (6.4 -17.6)	11.5 (5.5 - 15.6)	11 (7.4 -18.4)	0.296
Platelets,x10^3^/mm^3^, mean (SD) ↑	327.0 (132.4)	323.5 (133.3)	330.2 (132.8)	0.803
Hemoglobin, g/dL, median (IQR)	12.4 (11.3 - 13.4)	12.4 (11.1 -13.4)	12.4 (11.3- 13.4)	0.430
Hematocrit, median (IQR)	39.2 (36.6 - 42.4)	38.8 (36.2 - 42.3)	39.4 (37.7 - 42.5)	0.199
INR	1.1 (1 - 1.2)	1.0 (1 - 1.2)	1.1 (1 -1.3)	0.220
Blood glucose, mg/dL, median (IQR)↑	170 (129.5 - 208.5)	159 (125 -208)	177 (143 - 209)	0.340
ALT, U/L, median (IQR) ↑	60.3 (37.9 - 95.2)	54.4 (38.1 - 85)	66.8 (36.9 - 95.2)	0.527
AST, U/L, median (IQR) ↑	45.8 (22.7 - 70.1)	40.9 (22.4 -70.3)	47.3 (28.6 -66.7)	0.389
Total Cholesterol, mg/dL, mean (SD)	172.88 (55.7)	178.38 (70.0)	165.8 (33.6)	0.670
Direct bilirubin, mg/dL, median (IQR)	0.17 (0.12 - 0.31)	0.19 (0.12 - 0.28)	0.16 (0.12 - 0.33)	0.495
Indirect bilirubin, mg/dL, median (IQR)	0.16 (0.11 - 0.20)	0.13 (0.1 - 0.19)	0.19 (0.12 - 0.24)	0.059
Total bilirubin, mg/dL,median (IQR)	0.34 (0.25 - 0.55)	0.34 (0.24 -0.46)	0.34 (0.29 - 0.59)	0.416
Creatinine, mg/dL, median (IQR)	0.9 (0.8 - 1.1)	0.9 (0.8 -1.2)	0.9 (0.7 - 1.0)	0.264
Lactate Dehydrogenase,U/L, median (IQR)	422.5 (245 - 772)	375 (198 - 537)	626 (280 - 892)	0.161
Creatine Kinase, U/L,median (IQR)	69.6 (46.3 - 155.3)	68.7 (45.6 - 155.3)	71.9 (146.5 - 48.8)	0.657
Urea, mg/dL median (IQR)	35.5 (24.4 - 53.3)	36.5 (26.5 - 57.2)	33.5 (23.1 - 49.6)	0.571
Ck-MB, U/L, median (IQR)	20.4 (16.3 - 29.4)	20.4 (15.6 - 32.9)	19.8 (17.3 -26.9)	0.900
C-reactive protein, mg/dL, median (IQR)	79.5 (33.6 - 138.9)	90.1 (32.6 - 138.9)	75.2 (37.6 -139.5)	0.948
> 100 (abnormal)	34/93 (36.6%)	17/45 (37.8%)	17/48 (35.4%)	
D-dimer, ng/mL, median (IQR) ↑	1048.9 (458 - 3192.1)	1251.8 (458 - 3371.2)	799.5 (442.8 -3192.1)	0.596
≥ 500 (abnormal)	35/49 (71.4%)	17/26 (65.4%)	18/23 (78.3%)	0.833
Serum ferritin, ng/L, median (IQR)	717 (377 - 1180)	681 (321 - 1200)	780 (415 - 1180)	0.759
IL-6, pg/ml median (IQR) ↑	35.5 (7.6 - 127.1)	32.3 (11.1 -127.1)	46.0 (7.6 -126.8)	0.949

**↑,** increased; **↓,** decreased compared to hospital reference; IQR, interquartile range; qSOFA, rapid sequential organ failure assessment; INR, International Normalized Ratio (prothrombin time); ALT, alanine aminotransferase; AST, aspartate aminotransferase; Ck-MB, creatinine kinase MB.

To compare the differences in the hematological and biochemical profile of the patients after the intervention, data from D7 were compared to that at D1 (**Table 2**). It was observed that there were significant differences between the D1 and D7 results for hemoglobin and hematocrit (p=0.0162 and p=0.0166, respectively) in the placebo group. Patients who received treatment with MP showed a significant drop in C-reactive protein (CRP) levels on D7 compared to D1 (p=0.042) (**Table 2**). Although deaths were reported in both groups at D7, the numbers were not significantly different (p=0.218 and p=> 0.999, in Placebo and MP, respectively).

**Table 2:** Comparison between D1 (pre-treatment) and D7 (approximately 2 days post treatment) patient clinical and lab findings for each of the treatment groups.

	Placebo				MP			
Findings	Total (N=104)	D1 (n=52)	D7 (n=52)	p-value	Total (N=98)	D1 (n=49)	D7 (n=49)	p-value
Died				0.218				>0.999
No	96/104 (92.3%)	52/52 (100.0%)	44/52 (84.6%)		97/98 (99.0%)	49/49 (100.0%)	48/49 (98.0%)	
Yes	2/104 (1.9%)	0/52 (0.0%)	2/52 (3.8%)		1/98 (1.0%)	0/49 (0.0%)	1/49 (2.0%)	
ICU				0.283				0.299
No	65/104 (62.5%)	45/52 (86.5%)	20/52 (38.5%)		71/98 (72.4%)	44/49 (89.8%)	27/49 (55.1%)	
Yes	13/104 (12.5%)	7/52 (13.5%)	6/52 (11.5%)		11/98 (11.2%)	5/49 (10.2%)	6/49 (12.2%)	
Intubated				0.283				0.502
No	65/104 (62.5%)	45/52 (86.5%)	20/52 (38.5%)		72/98 (73.5%)	44/49 (89.8%)	28/49 (57.1%)	
Yes	13/104 (12.5%)	7/52 (13.5%)	6/52 (11.5%)		10/98 (10.2%)	5/49 (10.2%)	5/49 (10.2%)	
Glasgow coma scale				0.734				0.288
3	14/104 (13.5%)	8/52 (15.4%)	6/52 (11.5%)		9/98 (9.2%)	4/49 (8.2%)	5/49 (10.2%)	
13	1/104 (1.0%)	1/52 (1.9%)	0/52 (0.0%)		4/98 (4.1%)	2/49 (4.1%)	2/49 (4.1%)	
14	2/104 (1.9%)	2/52 (3.8%)	0/52 (0.0%)		4/98 (4.1%)	4/49 (8.2%)	0/49 (0.0%)	
15	62/104 (59.6%)	41/52 (78.8%)	21/52 (40.4%)		67/98 (68.4%)	39/49 (79.6%)	28/49 (57.1%)	
Hemoglobin, g/dL, median (IQR)	12.0 (11.1-13.4)	12.4 (11.3-13.4)	11.1 (10.5 -11.5)	0.0162	11.9 (11.2-13.1)	12.4 (11.1-13.4)	11.9 (11.4-12.7)	0.3417
Leucocytes,× 10^3^/mm^3^, median (IQR)	9.7 (7.0-13.6)	9.6 (7.1-13.0)	9.9 (6.1-17.5)	0.6403	10.5 (8.1-14.8)	9.5 (8.0-14.8)	12.7 (8.7-15.6)	0.3013
Lymphocytes, %, median (IQR	10.9 (6.9-18.6)	11.0 (7.4-18.4)	10.8 (6.9-18.9)	0.8348	11.1 (5.2-17.7)	11.5 (5.5-15.6)	9.5 (4.4-22.6)	0.8078
Neutrophils, %, median (IQR)	82.5 (72.1-87.6)	82.5 (72.1-87.4)	82.4 (72.6 -88.8)	0.9335	82.2 (72.4-90.2)	82.2 (75.4-89.2)	75.1 (66.4-91.1)	0.3421
Hematocrit, median (IQR)	39.0 (36.3-42.3)	39.4 (37.7-42.5)	35.6 (32.6 -38.7)	0.0166	38.7 (35.9-42.1)	38.8 (36.2-42.3)	38.2 (35.8-40.4)	0.5381
Platelets,x10^3^/mm^3^, mean (SD)	324.9 (134.2)	330.2 (132.8)	304.2 (142.9)	0.5380	323.4 (135.8)	323.4 (133.3)	323.3 (146.9)	0.9969
INR	1.1 (1.1-1.3)	1.1 (1.0-1.3)	1.1 (1.1-1.1)	0.5764	1.0 (1.0-1.1)	1.0 (1.0-1.1)	1.1 (1.1-1.3)	0.0887
ALT, U/L, median (IQR)	65.9 (36.9-95.2)	66.8 (36.9-95.2)	46.5 (44.3-68.0)	0.5346	54.5 (38.5-91.7)	54.4 (38.1-85.0)	78.3 (39.0-161.0)	0.5789
AST, U/L, median (IQR)	47.3 (28.6-69.4)	47.3 (28.6-66.7)	54.9 (37.7-69.4)	0.6538	36.2 (21.7-65.0)	40.9 (22.4-70.3)	28.4 (18.9-59.8)	0.5396
Direct bilirubin, mg/dL, median (IQR)	0.2 (0.1-0.3)	0.2 (0.1-0.3)	0.3 (0.1-0.6)	0.8093	0.2 (0.1-0.3)	0.2 (0.1-0.3)	0.2 (0.2-0.3)	0.3611
Indirect bilirubin, mg/dL, median (IQR)	0.2 (0.1-0.3)	0.2 (0.1-0.2)	0.3 (0.1-0.3)	0.8013	0.1 (0.1-0.2)	0.1 (0.1-0.2)	0.2 (0.2-0.3)	0.1608
Total bilirubin, mg/dL, median (IQR)	0.3 (0.3-0.6)	0.3 (0.3-0.6)	0.4 (0.4-0.9)	0.2587	0.4 (0.2-0.6)	0.3 (0.2-0.5)	0.4 (0.4-0.6)	0.3794
Blood glucose, mg/dL, median (IQR)	174.0 (143.0-204.0)	177.0 (143.0-209.0)	170.5 (139.5-202.5)	0.8740	154.0 (124.0-226.0)	159.0 (125.0-208.0)	150.0 (119.0-266.0)	0.8095
Creatinine, mg/dL, median (IQR)	0.9 (0.7-1.1)	0.9 (0.7-1.0)	1.1 (0.7-2.1)	0.2072	1.0 (0.8-1.1)	0.9 (0.8-1.2)	1.0 (0.7-1.1)	0.8658
Urea, mg/dL, median (IQR)	35.5 (23.5-51.7)	33.5 (23.1-49.6)	42.8 (30.0-87.0)	0.1649	37.5 (27.6-59.0)	36.5 (26.5-57.2)	45.4 (34.6-61.4)	0.2670
LDH, U/L, median (IQR)	371.0 (241.0-772.0)	626.0 (280.0-892.0)	255.0 (207.0-329.0)	0.1048	375.0 (198.0-485.0)	375.0 (198.0-537.0)	309.5 (195.0-406.5)	0.4654
Creatine Kinase, U/L, median (IQR)	66.0 (44.2-161.9)	71.8 (48.8-146.4)	51.4 (38.7-168.8)	0.8753	60.3 (34.7-113.7)	68.7 (45.6-155.3)	46.5 (29.5-106.5)	0.1537
Ck-MB, U/L, median (IQR)	19.7 (18.0-28.7)	19.8 (17.3-26.9)	19.7 (18.0-28.7)	0.6525	20.4 (16.9-31.1)	20.4 (15.6-32.9)	20.5 (17.5-25.5)	0.9298
D-dímer, ng/mL, median (IQR)	1048.9 (471.1-3592.5)	799.6 (442.8-3192.1)	3953.0 (2496.0-7806.2)	0.0857	1441.9 (503.9-3523.9)	1251.8 (458.0-3371.2)	3304.0 (1872.0-5422.1)	0.2748
CRP, mg/dL, median (IQR)	75.3 (33.6-168.0)	75.2 (37.5-139.3)	105.0 (9.2-197.3)	0.9158	71.8 (17.6-125.2)	90.1 (32.6-138.9)	21.6 (9.6-72.2)	0.0420
Sodium, mmol/mL, median (IQR)	142.0 (138.2-144.0)	142.0 (139.0-144.0)	142.0 (137.4-147.7)	0.6149	141.0 (139.0-143.0)	141.0 (139.0-142.7)	142.0 (139.0-147.0)	0.2021
Potassium, mmol/mL, median (IQR)	4.3 (4.0-4.9)	4.3 (3.9-4.7)	4.8 (4.4-5.3)	0.0515	4.3 (3.9-4.6)	4.2 (3.8-4.5)	4.7 (3.9-5.4)	0.1085

ICU, Intensive care unit; IQR, Inter quartile range; ALT, alanine aminotransferase; AST, aspartate aminotransferase; INR, International Normalized Ratio (prothrombin time); LDH, Lactate dehydrogenase; Ck-MB, creatinine kinase MB, CRP, C-reactive protein.

When compared to the Placebo group, the MP group’s blood hemogram profile improved. However, these differences were not statistically significant (**Table 3**).

**Table 3:** A between-groups comparison of the lab hematological and biochemistry findings at D1 and D7.

Laboratory Findings	D1 (Pre-treatment)				D7			
	Total (N=101)	Placebo (n=52)	MP (n=49)	p-value	Total (N=101)	Placebo (n=52)	MP (n=49)	p-value
Hemoglobin, g/dL, median (IQR)	12.4 (11.3-13.4)	12.4 (11.3-13.4)	12.4 (11.1-13.4)	0.4296	11.5 (10.5-12.6)	11.1 (10.5-11.5)	11.9 (11.4-12.7)	0.2495
Leucocytes,× 10^3^/mm^3^, median (IQR)	9.6 (7.7-13.3)	9.6 (7.1-13.0)	9.5 (8.0-14.8)	0.4639	12.1 (7.1-16.2)	9.9 (6.1-17.5)	12.7 (8.7-15.6)	0.7220
Lymphocytes, %, median (IQR)	11.3 (6.4-17.6)	11.0 (7.4-18.4)	11.5 (5.5-15.6)	0.2958	10.2 (5.3-21.8)	10.8 (6.9-18.9)	9.5 (4.4-22.6)	0.4898
Neutrophils, %, median (IQR)	82.3 (72.6-88.4)	82.5 (72.1-87.4)	82.2 (75.4-89.2)	0.2421	78.8 (66.7-89.3)	82.4 (72.6-88.8)	75.1 (66.4-91.1)	0.8835
Hematocrit, median (IQR)	39.2 (36.6-42.4)	39.4 (37.7-42.5)	38.8 (36.2-42.3)	0.1992	37.0 (32.6-40.3)	35.6 (32.6-38.7)	38.2 (35.8-40.4)	0.3794
Platelets,x10^3^/mm^3^, mean (SD)	326.9 (132.4)	330.2 (132.8)	323.4 (133.3)	0.8030	315.0 (143.0)	304.2 (142.9)	323.3 (146.9)	0.7242
INR	1.1 (1.0-1.2)	1.1 (1.0-1.3)	1.0 (1.0-1.1)	0.0828	1.1 (1.1-1.1)	1.1 (1.1-1.1)	1.1 (1.1-1.3)	0.7213
ALT, U/L, median (IQR)	60.3 (37.9-95.2)	66.8 (36.9-95.2)	54.4 (38.1-85.0)	0.5267	61.0 (41.6-109.1)	46.5 (44.3-68.0)	78.3 (39.0-161.0)	0.4649
AST, U/L, median (IQR)	45.8 (22.7-70.1)	47.3 (28.6-66.7)	40.9 (22.4-70.3)	0.3894	40.7 (21.7-64.6)	54.9 (37.7-69.4)	28.4 (18.9-59.8)	0.2548
Direct bilirubin, mg/dL, median (IQR)	0.2 (0.1-0.3)	0.2 (0.1-0.3)	0.2 (0.1-0.3)	0.4950	0.3 (0.2-0.3)	0.3 (0.1-0.6)	0.2 (0.2-0.3)	0.6547
Indirect bilirubin, mg/dL, median (IQR)	0.2 (0.1-0.2)	0.2 (0.1-0.2)	0.1 (0.1-0.2)	0.0590	0.2 (0.2-0.3)	0.3 (0.1-0.3)	0.2 (0.2-0.3)	>0.9999
Total bilirubin, mg/dL, median (IQR)	0.3 (0.3-0.6)	0.3 (0.3-0.6)	0.3 (0.2-0.5)	0.4155	0.4 (0.4-0.6)	0.4 (0.4-0.9)	0.4 (0.4-0.6)	0.8815
Blood glucose, mg/dL, median (IQR)	170.0 (129.5-208.5)	177.0 (143.0-209.0)	159.0 (125.0-208.0)	0.3397	164.0 (120.0-213.0)	170.5 (139.5-202.5)	150.0 (119.0-266.0)	0.5123
Creatinine, mg/dL, median (IQR)	0.9 (0.8-1.1)	0.9 (0.7-1.0)	0.9 (0.8-1.2)	0.2636	1.0 (0.7-1.2)	1.1 (0.7-2.1)	1.0 (0.7-1.1)	0.3167
Urea, mg/dL, median (IQR)	35.5 (24.4-53.3)	33.5 (23.1-49.6)	36.5 (26.5-57.2)	0.5714	45.4 (32.1-73.2)	42.8 (30.0-87.0)	45.4 (34.6-61.4)	0.8030
Lactate Dehydrogenase, U/L, median (IQR)	422.5 (245.0-772.0)	626.0 (280.0-892.0)	375.0 (198.0-537.0)	0.1611	255.0 (202.5-388.0)	255.0 (207.0-329.0)	309.5 (195.0-406.5)	0.7728
Creatine Kinase, U/L, median (IQR)	69.5 (46.3-155.3)	71.8 (48.8-146.4)	68.7 (45.6-155.3)	0.6565	51.0 (36.8-121.0)	51.4 (38.7-168.8)	46.5 (29.5-106.5)	0.1704
Ck-MB, U/L, median (IQR)	20.4 (16.3-29.4)	19.8 (17.3-26.9)	20.4 (15.6-32.9)	0.8996	20.4 (17.5-28.7)	19.7 (18.0-28.7)	20.5 (17.5-25.5)	>0.9999
D-dímer, ng/mL, median (IQR)	1048.9 (458.0-3192.1)	799.6 (442.8-3192.1)	1251.8 (458.0-3371.2)	0.5955	3523.9 (2496.0-7320.2)	3953.0 (2496.0-7806.2)	3304.0 (1872.0-5422.1)	0.4795
CRP, mg/dL, median (IQR)	79.5 (33.6-138.9)	75.2 (37.5-139.3)	90.1 (32.6-138.9)	0.9479	26.9 (9.2-152.0)	105.0 (9.2-197.3)	21.6 (9.6-72.2)	0.3347
Sodium, mmol/L, median (IQR)	141.0 (139.0-143.0)	142.0 (139.0-144.0)	141.0 (139.0-142.7)	0.4319	142.0 (137.4-147.7)	142.0 (137.4-147.7)	142.0 (139.0-47.0)	0.8842
Potassium,mmol/L, median (IQR)	4.3 (3.9-4.5)	4.3 (3.9-4.7)	4.2 (3.8-4.5)	0.5474	4.8 (4.0-5.4)	4.8 (4.4-5.3)	4.7 (3.9-5.4)	0.8271

### 3.2 Injury biomarker profile in the MP and placebo group

On admission, biomarker levels were comparable between the two groups, suggesting that the effect of COVID-19 infection on patients was similar. No significant differences were observed between the two groups at D1 *vs.* D7 and D14, except for HMGB1, which was significantly lower in the placebo group than MP group on D7 (p = 0.0447) (**Table 4**). Baseline and follow-up values of myoglobin, NTproBNP, troponin I, FABP were comparable between groups on different follow-up days (p>0.05).

**Table 4:** Tissue injury biomarkers of COVID-19 patients on either MP or placebo treatment.

Analytes *	Day	n (P; M)	Placebo	MP	P-value
**HMGB1 (ng/ml)**	D1	51; 47	205.99 (105.24-411.51)	193.05 (65.19-439.11)	0.6213
	D7	21;27	117.31 (74.29-250.25)	250.25 (127.94-420.51)	0.0448
	D14	7; 7	57.2 (1.93-74.29)	88.43 (0.93-239.66)	0.6888
**MYGBN (ng/ml)**	D1	37; 41	95.59 (68.61-162.26)	128.77 (78.11-214.79)	0.2056
	D7	21; 30	88.47 (62.73 -176.52)	100.54 (71.45-132.85)	0.4908
	D14	6; 7	145.3 (61.00 - 755.20)	82.62 (62.53-429.85)	0.8864
**FABP3 (pg/ml)**	D1	46; 47	4486.49 (2398.69-6866.58)	5244.93 (2986.85-7396.27)	0.2592
	D7	22; 25	4355.97 (2761.17- 2743.28)	6157 (4527-11993)	0.1396
	D14	6; 10	10333.09 (3740.49-25014.41)	22003.67 (3150.97-75115.13)	0.8749
**TnI (pg/ml)**	D1	32; 34	1199.58 (663.32-3816.48)	2422.04 (1212.36-5676.05)	0.0543
	D7	13; 27	955.13 (762.44-2368.37)	2075.55 (736.15-5342.87)	0.4615
	D14	6; 9	1278.25 (707.31-2815.59)	2287.79 (900.86-8548.62)	0.3458
**NTproBNP (pg/ml)**	D1	21; 23	128.94 (63.55-253.80)	90.44 (16.59-174.88)	0.2258
	D7	11; 15	231.66 (83.82-412.23)	89.87 (16.59-250.62)	0.1764
	D14	5; 8	139.27 (59.95-361.30)	73.4 (20.35-165.3)	0.3714

*Median (IQR); MP, methylprednisolone treated group; HMGB1, high-mobility group box-1; MYGBN, Myoglobin; FABP3, Heart-type fatty-acid-binding protein 3; TnI, Troponin I; NTproBNP, N-terminal pro-brain natriuretic peptide.

A longitudinal analysis indicated that HMGB1 levels in the placebo group significantly decreased with time from a median of 205.99 ng/mL on D1 to 57.2 ng/mL on D14 (p=0.0115) (**Table 5**). In contrast, HMGB1 levels in the MP group were similar during follow-up (p = 0.1856). On the other hand, myoglobin in the placebo group had an increasing tendency from D1 to D14 (95.59 to 145.3 ng/ml), while in the MP group it decreased in the same period (128.77 to 82.62 ng/ml), but without significant difference (p>0.05). FABP3 levels in the placebo group seemed to stabilize between D1 and D7 before increasing on D14 (4486.49 to 6522 pg/mL) (p=0.4171), while in the MP group this biomarker steadily increased from D1 to D14 (5244.93 to 22003.67 pg/ml) (p=0.0509).

**Table 5:** Longitudinal evaluation of injury biomarkers in COVID-19 patients treated with MP and placebo over a 14-day period.

Analytes	Group	D1	D7	D14	P-value
**HMGB1 (ng/ml)**	Placebo	205.99 (105.24-411.51)	117.31 (74.29-250.25)	57.2 (1.93-74.29)	0.0115
	MP	193.05 (65.19-439.11)	250.25 (127.94-420.51)	88.43 (0.93-239.66)	0.1856
**MYGBN (ng/ml)**	Placebo	95.59 (68.61-162.26)	88.47 (62.73-176.52)	145.3 (64.77-540.72)	0.9111
	MP	128.77 (78.11-214.79)	100.54 (71.45-132.85)	82.62 (62.53-429.85)	0.6222
**FABP3 (pg/ml)**	Placebo	4486.49 (2398.69-6866.58)	4355.97 (2761.17-12743.28)	6522 (3333-1959)	0.4171
	MP	5244.93 (2986.85 - 7396.27)	6157 (4527-11993)	22003.67 (3150.97-75115.13)	0.0509
**TnI (pg/ml)**	Placebo	1199.58 (663.32-3816.48)	955.13 (762.44-2368.37)	1278.25 (707.31-2815.59)	0.9604
	MP	2422.04 (1212.36-5676.05)	2075.55 (736.15-5342.87)	2287.79 (900.86-8548.62)	0.5920
**NTproBNP (pg/ml)**	Placebo	128.94 (82.42-249.27)	231.66 (83.82-412.23)	139.27 (116.51-357.08)	0.5704
	MP	90.44 (16.59-174.88)	89.87 (16.59-250.62)	73.4 (20.35-147.66)	0.8757

TnI levels in both groups were constant during follow-up. For NTproBNP, mean levels in the placebo group increased at D7 before falling at D14 (128.94 to 231.66 to 139.27 pg/ml at D14). In the MP group, NTproBNP remained constant on D1 and D7, approximately 90 pg/ml, before a slight drop on D14 to 73.4 pg/ml. Again, these observed changes were not significant (p>0.05) (**Table 5**).

### 3.3 The serum cytokine and chemokine of COVID-19 patients

**Figure 1:**
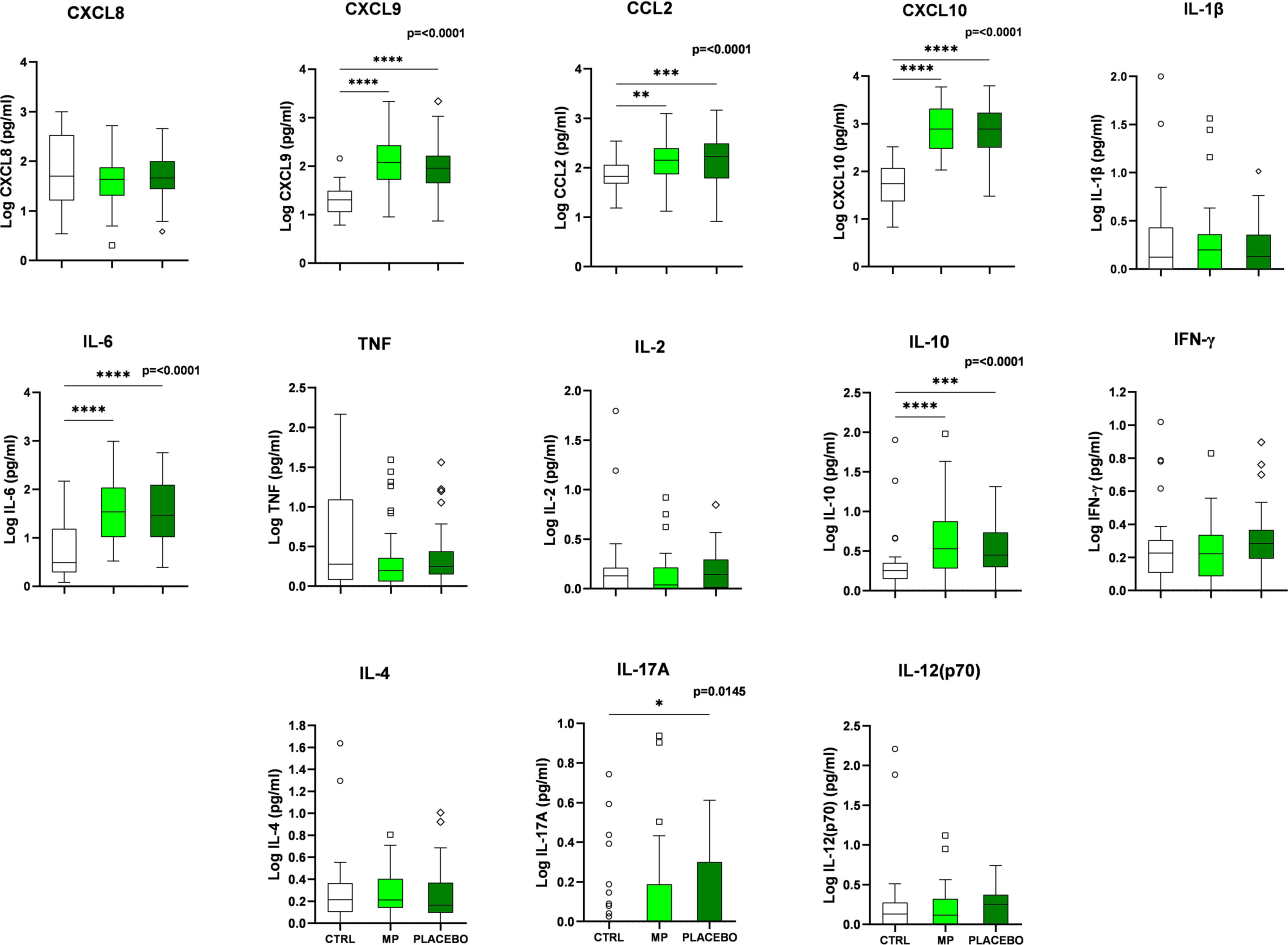
Serum cytokine and chemokine levels in COVID-19 infected patients compared to the healthy participants (negative controls) at baseline (D1). COVID-19 negative controls (CTRL), Methylprednisolone treated patients (MP), Placebo/normal saline treated patients (PLACEBO), Interleukin-2 (IL-2), Interleukin-4 (IL-4), Interleukin-8 (IL-8), Interleukin-1β (IL-1β), Interleukin-6 (IL-6), Interleukin-10 (IL-10), Interleukin-12p70 (IL-12p70), Interleukin-17A (IL-17A),Tumor Necrosis Factor (TNF), Interferon-γ (IFN-γ), Monokine-induced by Interferon-γ (MIG/CXCL9), Monocyte Chemoattractant Protein-1 (MCP-1/CCL2), and Interferon-γ-induced Protein-10 (IP-10/CXCL10). *p < 0.05, **p < 0.01, ***p < 0.001, and ****p < 0.0001.

**Figure 1** shows the serum cytokine and chemokine profile of the COVID-19 patients at the D1 baseline. When compared to the control group at D1, levels of IL-10, IL-17A, IL-6, MIG/CXCL9, MCP-1/CCL2 and IP-10/CXCL10 were significantly elevated in the patients. A comparison between the MP and placebo groups at D7 revealed that the chemokines MIG/CXCL9 and IP-10/CXCL10 were significantly elevated in the Placebo group. No significant differences were observed in the serum immunological molecules at D14 (**Figure 1_Supplementary Files**).

When comparing the baseline cytokines/chemokines results (D1) of the controls and the MP group as seen in **Figure 1**, six immune mediators were significantly high. This number declined during the D7 analysis (**Supplementary Figure 1**). This decline occurred over time after the MP-intervention. Some of these immune molecules responded quickly, and others slowly, relative to the baseline levels.

### 3.4 Longitudinal profile of the immune modulators after treatment of COVID-19 patients

**Figure 2:**
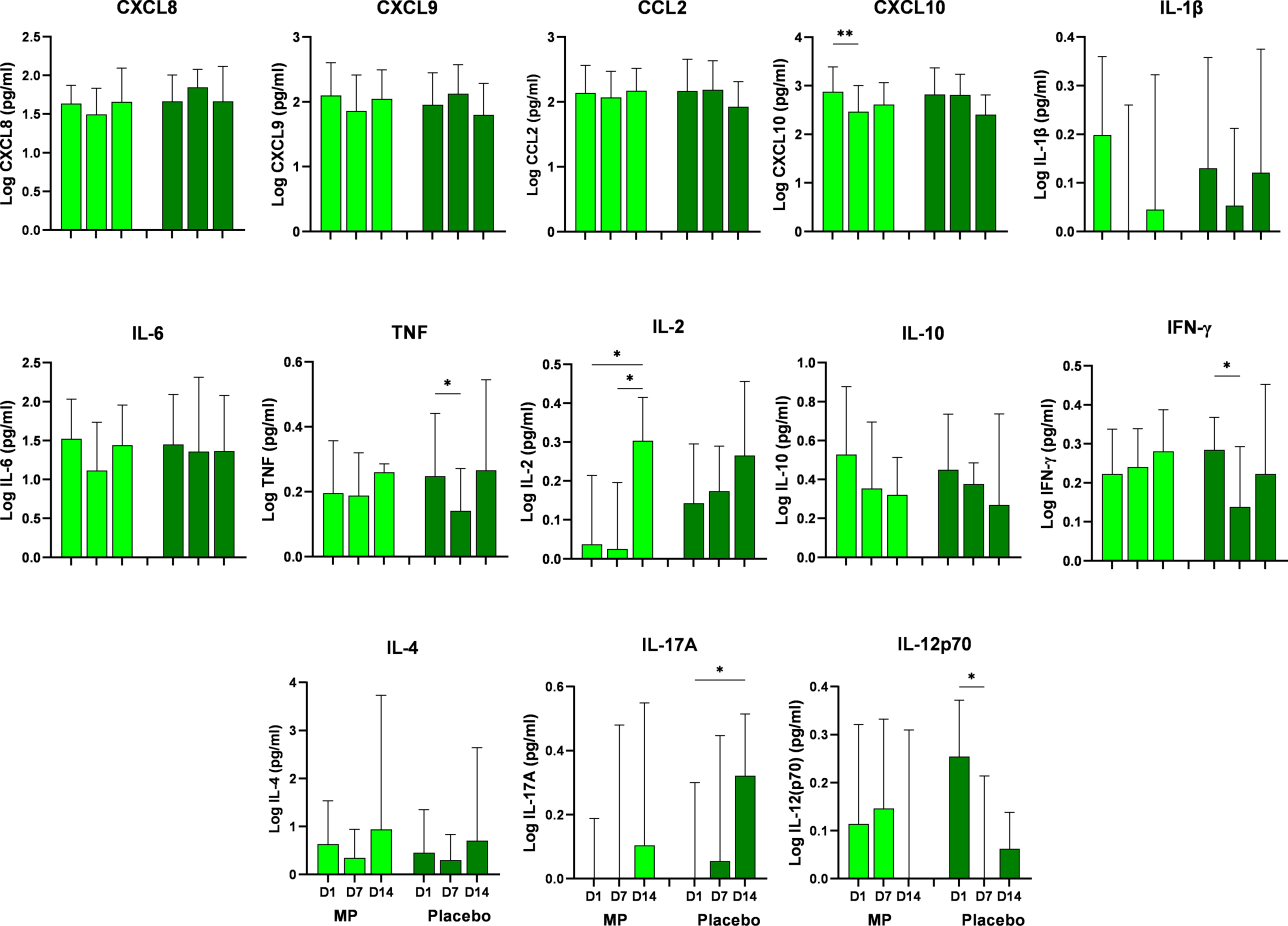
Serum concentrations of chemokines and cytokines in treated COVID-19 patients (MP-treated and Placebo groups) during the follow up (D1 to D14). Statistical difference between follow up times was considered significant when p < 0.05 (^*)^. Statistical analysis was performed using the Kruskal-Wallis test followed by Dunn’s posttest or the Ordinary ANOVA test with Tukey’s post-test where necessary. Methylprednisolone treated patients (MP), Placebo/normal saline treated patients (Placebo), Interleukin-2 (IL-2), Interleukin-4 (IL-4), Interleukin-8 (IL-8), Interleukin-1β (IL-1β), Interleukin-6 (IL-6), Interleukin-10 (IL-10), Interleukin-12p70 (IL-12p70), Interleukin-17A (IL-17A),Tumor Necrosis Factor (TNF), Interferon-γ (IFN-γ), Monokine-induced by Interferon-γ (MIG/CXCL9), Monocyte Chemoattractant Protein-1 (MCP-1/CCL2), and Interferon-γ-induced Protein-10 (IP-10/CXCL10), D1- day 1, D7- day 7, D14 - day 14. *p < 0.05, **p < 0.01.

**Figure 2** depicts the levels of cytokines and chemokines in the MP and placebo groups during follow-up. The MP group demonstrated a progressive increase in IL-2, IFN-γ, IL-17A and IL-12p70 concentrations towards D14, but only IL-2 had a significant increase by D14.The placebo group IL-2 and IL-17A levels followed the same progressive pattern (**Figure 2**), with only IL-17A increasing significantly by D14. On the other hand, concentrations of IL-1β, IL-10 and IP-10 (CXCL10) in MP group and IFN-γ, IL-12p70, IL-10, MCP-1 (CCL2), MIG (CXCL9) and IP-10 (CXCL10) in the placebo group decreased longitudinally (**Figure 2**).

A longitudinal analysis of cytokine and chemokine levels from D1 to D14 showed significant increase in IL-2 (between D1and D14, and D7 and D14), and significant decrease between D1 and D7 in IP-10 (CXCL10) levels within the MP group. Significant changes were observed in the profile of IFN-γ, TNF and IL-17A within the Placebo group, mostly occurring between D1 and D7 (**Figure 2**). Specifically, these significant changes in the Placebo group were a decrease in IFN-γ, TNF, and IL-12p70 where all decreased by D7 and an increase in IL-17A by D14.

### 3.5 COVID-19 patients have complex biomarker networks with moderate to strong interactions in the circulating serum cytokines/chemokines of the MP-treated group

**Figure 3:**
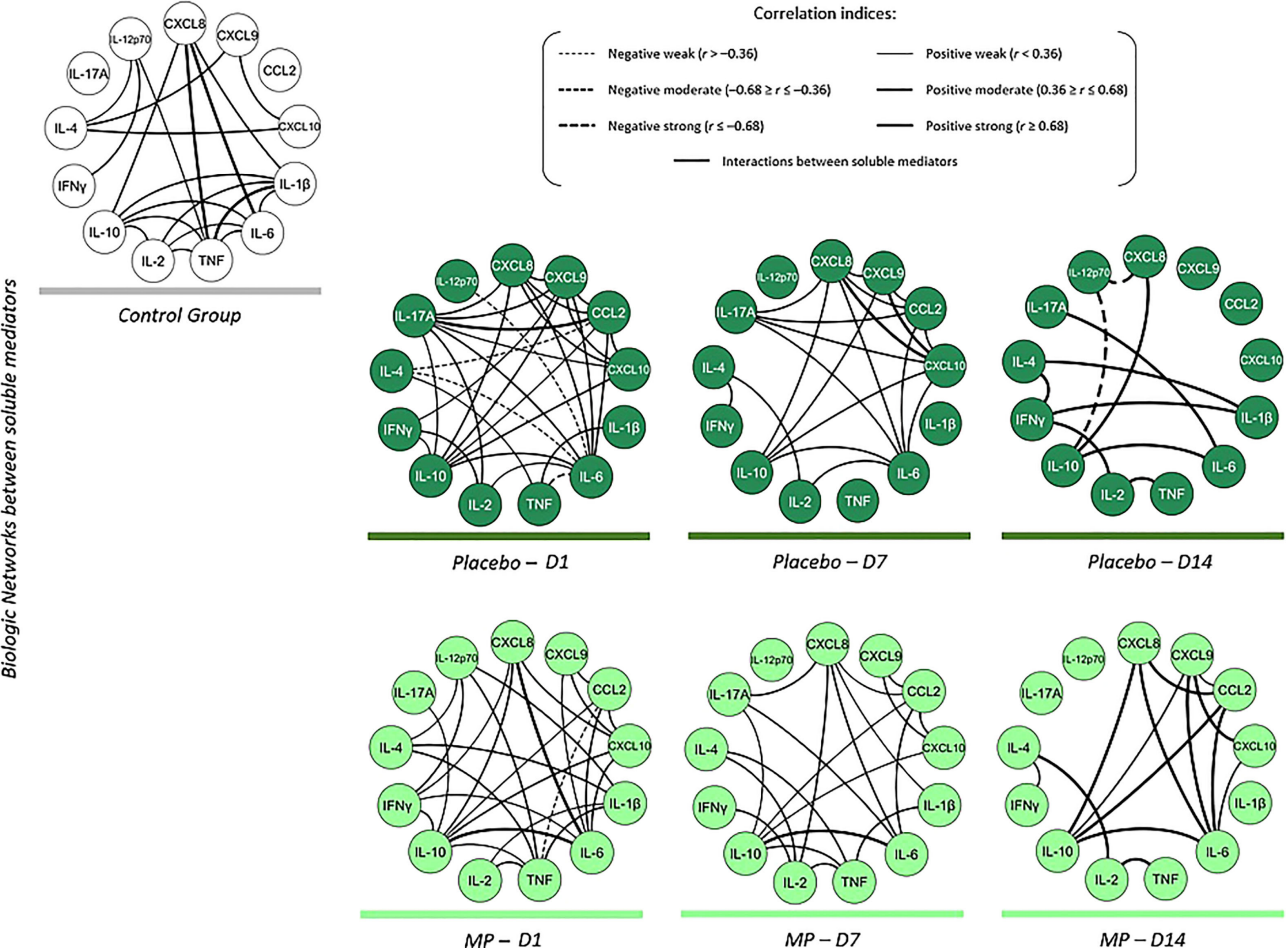
Immune mediator networks show the interactions between the groups in the follow-up of the study. Color codes were used to identify the different study groups as follows: Control group (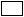), Placebo (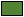) and MP group (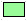). The different line sizes and types demonstrate the interrelationships between the chemokines and cytokines circulating in the peripheral blood from the different study groups. Dashed lines between molecules indicate a negative correlation, while solid lines indicate a positive correlation. The thickness of these indicates the strength of the correlation. The correlation index (r) used to categorize the strength of the correlation as weak (r ≤ 0.35), moderate (r≥0.36 to r ≤ 0.67) or strong (r≥0.68).

COVID-19 patients had an immunological marker interaction network different from the healthy non-COVID-19 controls, as shown in **Figure 3**. The control group showed strong correlations between TNF and IL-1β, CXCL8/IL-8 and IL-6, and CXCL8/IL-8 and TNF. On D1 (prior to intervention), weak levels of IL-10 regulation for cytokines and modest chemokine modulation by IL-10 response were detected in the placebo group, unlike the MP group, where strong positive correlations between IL-10 with IL-6 and IL-8/CXCL8 with IL-6 were observed. The two infected groups demonstrated numerous chemokine–cytokine (IL-10, IL-6 and IL-17A) correlations; the healthy controls had more of cytokine-cytokine relationships (IL-10/IL-6 with IL-1β, TNF, and IL-2).

After the intervention, patients treated with MP showed moderately increased positive correlations of chemokines with other immune mediators during clinical follow-up (D7). A clear inflammatory response was revealed at D7 within the placebo treated patients. Of interest in the placebo group was the strong chemoattractant activities advanced by the strong correlations between IP-10/CXCL10 and IL-8/CXCL8, and by IP-10/CXCL10 with IL-8 and MIG/CXCL9.

Contrary to the observations on D1 and D7, there was predominantly strong positive correlation between cytokines and chemokines on D14 in both treatment groups. MP-treated group also presented few moderate chemokine-cytokine interactions. The D14 placebo group interactions were characterized by substantial correlations between pro-inflammatory and regulating immune molecules.

## 4 Discussion

The complex immunopathological response following SARS-CoV-2 infection is responsible for varied disease pathogenesis and clinical manifestations. This immunopathological response arises from activated immune responses against the SARS-CoV-2, and uncontrolled inflammatory responses characterized by marked pro-inflammatory cytokine release in patients with severe COVID-19, leading to lymphopenia, lymphocyte dysfunction, and granulocyte and monocyte abnormalities. Knowing the effect of corticosteroid intervention on the host’s immune responses and injury biomarkers during COVID-19 infection would allow for an early assessment of the benefit and/or risk of this treatment against immunity- or virus-induced pathology, and evidence of crosstalk between injury and inflammation biomolecules. Currently, the success of corticosteroid treatment varies on dose, treatment time, stage of disease and patient factors.

Baseline clinical results revealed similar observations, particularly lower lymphocyte counts, elevated neutrophil counts, high platelet counts, high dimer readings and impaired liver function. These observations corresponded similar observations among COVID-19 patients in China (49–51).

The use of methylprednisolone was associated with a significant reduction in CRP levels on day 7. Torres et al. also observed that patients who received corticosteroids showed differences in the systemic anti-inflammatory effect measured by CRP on day 3, with variations related to the presence or absence of lymphopenia (52).

A comparison of the D1 and D7 laboratory findings revealed no significant differences in the hematological and biochemistry parameters. However, we found significant decline in hemoglobin and hematocrit levels in the Placebo group, agreeing with findings from previous studies that analyzed the worsening of the disease (53–59). Comparing the changes in the groups after the corticosteroid treatment, they were not significantly different in the placebo group. This can be explained by time lost (in days) between onset of symptoms until inclusion, time to treatment and follow-up considering that clinical parameters vary with disease severity, patient’s immunity status, and viral characteristics like incubation time, replication phases, and duration of shedding (60), and all groups were of severe cases.

The High-mobility group box 1 (HMGB1) is a multifunctional nuclear protein actively secreted by necrotic cells in response to pathogens or endogenous inflammatory stimuli, and can be passively released by damaged lung parenchyma cells, as in the case of COVID-19 (42). Al-kuraishy et al. summarized that high levels of HMGB1 are synonymous with disease severity, development of cytokine storm (CS), acute lung injury and acute distress syndrome (ARDS) (61), and it is a potential prognostic factor for severe COVID-19 and mortality. HMGB1 levels in our cohort at D1 were significantly elevated. Sivakorn et al. showed that HMGB1 levels at ICU admission were higher in patients with COVID-19 than in healthy subjects (42). Taken together, these findings underscore the potential crucial role of HMGB1 as a biomarker for COVID-19 disease severity.

Despite the MP intervention, HMGB1 levels in treated patients were still elevated. Although there was a significant difference in HMGB1 levels in the Placebo group between D1 and D14. However, Gougeon et al. (62) and Rubartelli and Lotze (63) argue that DAMPs, such as HMGB1, once released are limited spatially and temporally until they get oxidized, essentially reducing inflammation by minimizing prolonged stimulation of its targets (62, 63). The high levels of circulating HMGB1 in our MP-treated group could arise from its potent action as an alarmin, in an attempt to promote an effective immune response against the pathogen (especially Th1) or that the damage still present. Although, the corticoid has the action of suppressing this response, it is not necessarily succeeding in regenerating tissue or reversing the damage, in which the alarmin is still “giving the warning”. The observation in our study is in line with claims that circulating levels of HMGB1 are elevated for prolonged periods in severe infections such as sepsis and COVID-19 (64) further confirming its role as a late mediator of systemic inflammation.

Several drugs, and peptide-based and small-molecule HMGB1-targeted therapeutics can act as specific HMGB1 antagonists, improving inflammation, ameliorating tissue injury and improving survival (65, 66). In COVID-19 for instance, dexamethasone can potentially modulate the HMGB1 signaling pathway in infected patients (67). This may explain the differential effect between different corticosteroid treatments, since unlike dexamethasone, MP or hydrocortisone cannot modulate the HMGB1-induced inflammatory response (68). The lack of effect of MP therapy on HMGB1 levels as seen in the present investigation may be explained by disparity in the capacity of various corticosteroids to modify HMGB1. These findings suggest that MP mode of action did not support or promote an immediate dissipation of HMGB1 production and its effects in patients with COVID-19.

Myoglobin is an iron and oxygen-binding protein that is important in the storage of oxygen in skeletal and cardiac muscles. Likewise, myoglobin has a prognostic value as a marker of myocardial injury in patients with COVID-19 (69). Our findings are in line with observations of myoglobin as an important COVID-19 biomarker. Yu et al. demonstrated that myoglobin was a predictive factor for in-hospital deaths from COVID-19. Corticosteroids can decrease inflammatory responses and reduce immune-mediated damage as seen in COVID-19 (70). Although not significantly, methylprednisolone intervention in our COVID-19 cohort improved serum creatine kinase and myoglobin levels.

Fatty acid binding protein 3 (FABP3), also known as Heart fatty acid binding protein (HFAB), is a highly specific myocardial injury serum biomarker (71). This protein reversibly and non-covalently binds long-chain fatty acids to facilitate intracellular cytoplasmic transport of fatty acids. Recent studies found that elevated levels of FABP3 were associated with severe COVID-19 (72). Our cohort demonstrated normal levels of FABP3 (ranging from 1.6 ng/mL to 19 ng/mL) in accordance with several cardiovascular disease studies (73–78). In addition to the treatment, the observed normality may have been influenced by the >36h delay from the onset of symptoms to when patients sought treatment. Although not significant, FABP3 levels in MP group on D14 were twice as high as in placebo-treated patients.

Troponin I and N-terminus of brain natriuretic peptide prohormone (NT-proBNP) are specific for myocardial injury and their increase is influenced by the severity of the disease. In our study, it was observed no significant difference in Troponin I (TnI) levels between placebo and MP groups on days 1, 7 and 14. Our results were also in agreement with the observations of Samprathi and Jayashree (3) who note that TnI and NT-proBNP are increased in patients with COVID-19 and serve as important cardiac biomarkers (3).

Another study reported that corticosteroids may protect patients with pneumonia against myocardial injury and poor cardiovascular outcomes (79). This study however used the serum high-sensitivity troponin (hs-cTnT) and not TnI as an indicator of myocardial injury, and patients received either methylprednisolone (20-80 mg/d), betamethasone (4-8 mg/d) or prednisone (25-50 mg/d) unlike the biomarker and MP dose used in our study.

Gao et al. observed that NT-proBNP was significantly higher in COVID-19 fatal cases compared with non-severe (80). Like TnI, high NT-proBNP is significantly indicative of poor prognosis among patients with COVID-19 suggesting that NT-proBNP is associated with the severity of the infection, leading to death. Other studies have confirmed similar observations regarding TnI and NT-proBNP and in-hospital mortality (28, 33, 51). NT-proBNP is secreted in response to increased myocardial wall stress, such as ischemia. Although NT-proBNP levels in MP group declined over time, it was not significant. Overall, NT-proBNP levels do not appear to be significantly influenced by MP treatment, perhaps due to the 1-week interval between assessments, but the biomarker is useful for assessing severity and predicting mortality.

Compared with controls, SARS-CoV-2 infection triggered an elevation in anti-inflammatory cytokine IL-10 and pro-inflammatory cytokine IL-6 and IL-17A. In addition, a significant elevation in the chemokine profile particularly in monocyte chemoattractant protein-1 (MCP-1), Th1 monocyte chemokines induced by interferon gamma (MIG) and IFN-γ-10 inducible protein (IP-10).These observations were similar to those from SARS patients (81), and COVID-19 patients (82–84). Additionally, some of the inflammatory cytokines and chemokines elevated in our study have been associated with the cytokine storm that cause acute lung injury and amplified inflammatory response that is deleterious to other organs (83, 85). Further, after the viral infection, these cytokines also stimulate pathways that result in immune cell differentiation, and the movement of leukocytes to infection sites further aggravating the inflammation and tissue injury (1, 86).

When compared to the placebo group, MP appeared to modify IL-8/CXCL8, IL-17A, IL-1β, IL-6, MIG/CXCL9, MCP-1/CCL2, and IP-10/CXCL10 levels by day 2 after end of therapy (D7). MP may inhibit the synthesis of NF-κB, which is responsible for the stimulation of expression of genes that code for inflammatory molecules like TNF, IL1, MCP-1/CCL2 and IL-8/CXCL8 (87). Studies have observed that the use of corticosteroids reduces TNF-alpha and IL-6 levels by reducing the systemic inflammatory response in patients hospitalized for CAP (88).

On D7, MIG/CXCL9 and IP-10/CXCL10 were significantly reduced in the MP group than the placebo. These cytokines are responsible for chemotaxis, differentiation, multiplication and tissue extravasation of leukocytes. They are strongly induced by IFN-γ (89), suggesting that the corticosteroid intervention interrupted the subsequent chemotactic effects influenced by IFN-γ in COVID-19. On a similar note, the significant difference in MIG and IP-10, as at D7 between the two groups could also be indicative of the difference in disease severity existing after the intervention and follow-up. As noted by Yang et al., although some cytokines were elevated in non-severe patients, they were also significantly lower than in severely ill COVID-19 patients (82).

Longitudinal data from the present study indicate that MP had a reducing effect on IL-2, IL-10 and IP-10. A longitudinal analysis of the cytokine profile after MP treatment showed a steady increase in IFN-γ and a corresponding decline in IL-10 between D1 and D14. This can be attributed to the short 5-day course of treatment and the short half-life of the drug (90). We postulate that the relatively high longitudinal levels of IFN-γ, IL-8, IL-6, MCP-1, MIG and IP-10 could be due to delayed viral clearance, disease severity (91) and possibly high SARS-CoV-2 viral load.

There was an observed difference in correlations between controls and pretreated COVID-19. In the control group, there was an active IL-10-IL-6 balance as well as correlation between IL-8/CXCL8, IL-1β, and TNF with other mediators. Compared with the control group, there was extensive cytokine/chemokine correlations centered on IL-17A, IL-10, IL-6, CCL2, CXCL8, CXCL9 and CXCL10 and fewer correlations with IL-1β in the COVID-19 patients at baseline. In severe cases, through inflammatory mediators like IFN-α, IL-1β, IL-6, CCL2, CCL5 and others, COVID-19 causes an inflammatory process that if uncontrolled evolves into lung tissue injuries, systemic inflammations and pathogenesis, and organ failure (1). The inflammatory profile and mediator profile of this disease may vary from factors such as patient immunodeficiency status, age, weight, gender, among others (1, 9, 92).

The corticoid treatment saw more correlations associated with IL-10, IL-2, TNF and IL-1β. Although all the associations here were mainly moderate positive, our analysis revealed strong correlation between IL-10 and IL-6 in MP treated, compared to CXCL10 with CXCL8 and CXCL9 in the placebo group. A proposed mechanism of action of these natural hormones and their synthetic analogs is to block the synthesis and secretion of cytokines, including TNF and IL-1, and other inflammatory mediators, such as prostaglandins, reactive oxygen species, and nitric oxide, produced by macrophages and other inflammatory cells (87).

Corticosteroids block the response of neutrophils to damage tissues and inhibit the chemotaxis of monocytes and neutrophils to sites of inflammation. Furthermore, these drugs block T-cell activation through inhibition of cytokine releases, thus decreasing levels of interleukin IL1, IL-2, IL-6, and TNF-α. Corticosteroids’ anti-inflammatory and immunosuppressive effects are dose- and time-dependent (70). By day 14, the MP treated patients exhibited strong correlations between IL-1, IL-6, CCL2, CXCL9, and CXCL8. On the contrary, patients receiving placebo therapy had the correlations only between IL-10, IL-6, and IL-1β and with none in the chemokines. Strong but negative correlations, IL-12p70 with IL-10 and CXCL8, were noted in the Placebo group.

In addition to drugs, several other factors, like host factors, can influence the production and correlation of human cytokines (93). The host factors can be genetic or non-genetic host factors like age, obesity and gender. Though our cohort was homogenous in age, sex and body mass index, we cannot rule out the potential influence cytokine gene variants. Hoffmann et al. report that there is a wide inter-ethnic variability in cytokine gene variants’ frequencies (*IL-2, IL-6, IL-10, TNF, TGFB1*, and *IFN-γ*) (94). These host genetic background is a critical influencer in COVID-19 outcome (95). A review study observed that variants in cytokine genes such as *IL-1β, IL1R1, IL1RN, IL-6, IL-17A, FCGR2A*, and *TNF* may be associated with disease susceptibility and/or severity, cytokine storm, and/or COVID-19 complications like thrombosis (96). Although we did not examine the pharmacogenomics of MP metabolism and presence of cytokine variants in our cohort, we cannot rule out their impact on the cytokine/chemokine profile. We also hypothesize that other than the MP treatment, that the injury biomarkers profile in our cohort may have influenced the observed mediator-mediator interrelationships.

Our data demonstrated that serum biomarkers HMGB1, myoglobin, FABP3 and troponin I were significantly altered during the initial phase of COVID-19. At D7, the MP had ameliorated levels of myoglobin and FABP3. Analysis of cytokine at pretreatment showed that the COVID-19 positive patients had significantly elevated levels of IL-17A, IL-6, IL-10, MIG, MCP-1 and IP-10. At D7, the MIG and IP-10 levels in MP group were significantly lower than placebo group. The intervention also lowered the IL-17A, IL-6, IL-1β, MCP-1, and IL-8 levels though not significantly. The MIG/CXCL9 induces chemotaxis, promote differentiation and multiplication of leukocytes, and cause tissue extravasation whereas the IP-10/CXCL10 as an inflammatory chemokine mediates immune responses through the activation and recruitment of leukocytes such as T cells, eosinophils, monocytes and NK cells (97). The observations at D7 are suggestive of how MP relieves the COVID-19 induced inflammatory response. Longitudinally, MP therapy influenced the IP-10 and IL-2 profiles, and decreased the IL-1β, and IL-10 levels. Conversely, IFN-γ levels steadily increased following this treatment. Interestingly IL-2 and IL-17A increased significantly in the placebo group, while IL-12p70, IL-10 and IP-10 decreased with time. Finally, unlike the 10-day dexamethasone study (16), the overall usage of MP for 5 days was not, in theory, effective in improving patient prognosis (45), despite the alterations in the cytokine profile.

This study had some limitations. The longitudinal decrease in sample size during injury-associated biomarker analysis and the lack of healthy controls restricted a full appreciation of biomarker dynamics. The sample size limitation subsequently affected our ability do a correlation network between the biomarker and cytokine profiles. The time interval between sample collection and analysis may have influenced the cytokine and biomarker levels detected considering that cytokines degrade during long time storage. In addition, considering that we used data extracted from patient records, there was incomplete collection or recording of full laboratory test data at D14 follow up. Despite these limitations, the results presented here help get an understanding the influence corticosteroid intervention has on the cytokine and biomarker profile of COVID-19 patients.

## Conclusion

Overall, the use of high-daily dose methylprednisolone in severe COVID-19 management was beneficial to the immunological response, especially the pro-inflammatory cytokines, but limited on the injury biomarker profile. We theorize that the methylprednisolone effect the injury biomarker and cytokine profile could be dose-influenced because of the high daily dose used and treatment-time influenced considering the median 12 days delay from onset of symptoms to inclusion and start of the MP-treatment. We propose that more focused studies in future should explore influence of different doses, time to treatment, age, and sex postulates to evaluate how best to position and fortify methylprednisolone’s use in the management of inflammatory reactions associated with corona virus infections like SARS, MERS and COVID-19.

## Data availability statement

The original contributions presented in the study are included in the article/**Supplementary Material**. Further inquiries can be directed to the corresponding author.

## Ethics statement

The studies involving human participants were reviewed and approved by Fundação de Medicina Tropical Doutor Heitor Vieira Dourado. The patients/participants provided their written informed consent to participate in this study.

## Author contributions

VM, AC, WM, and GM conceived the study concept and designed the experiments. VM, RN, CM, AL, and AC performed the experiments, and data acquisition. VM, AS, BS, and JN analyzed and interpreted the data, and did data visualization. VS advised on the data analysis and interpretation. VM, RN, and JN and wrote the manuscript. AA, AC, FV, ML, LG, VS, WM, and GM critically revised the manuscript and results. MB, AC, ML, WM, and GM provided administrative, technical, and material support and supervised the study. All authors contributed to the article and approved the submitted version.
