# Supplementary figures and images for: Temporal patterns of cytokine and injury biomarkers in hospitalized COVID-19 patients treated with methylprednisolone
Source: Front Immunol. 2023 Aug 16;14:1229611. doi: 10.3389/fimmu.2023.1229611 (PMC10468998; doi:10.3389/fimmu.2023.1229611)

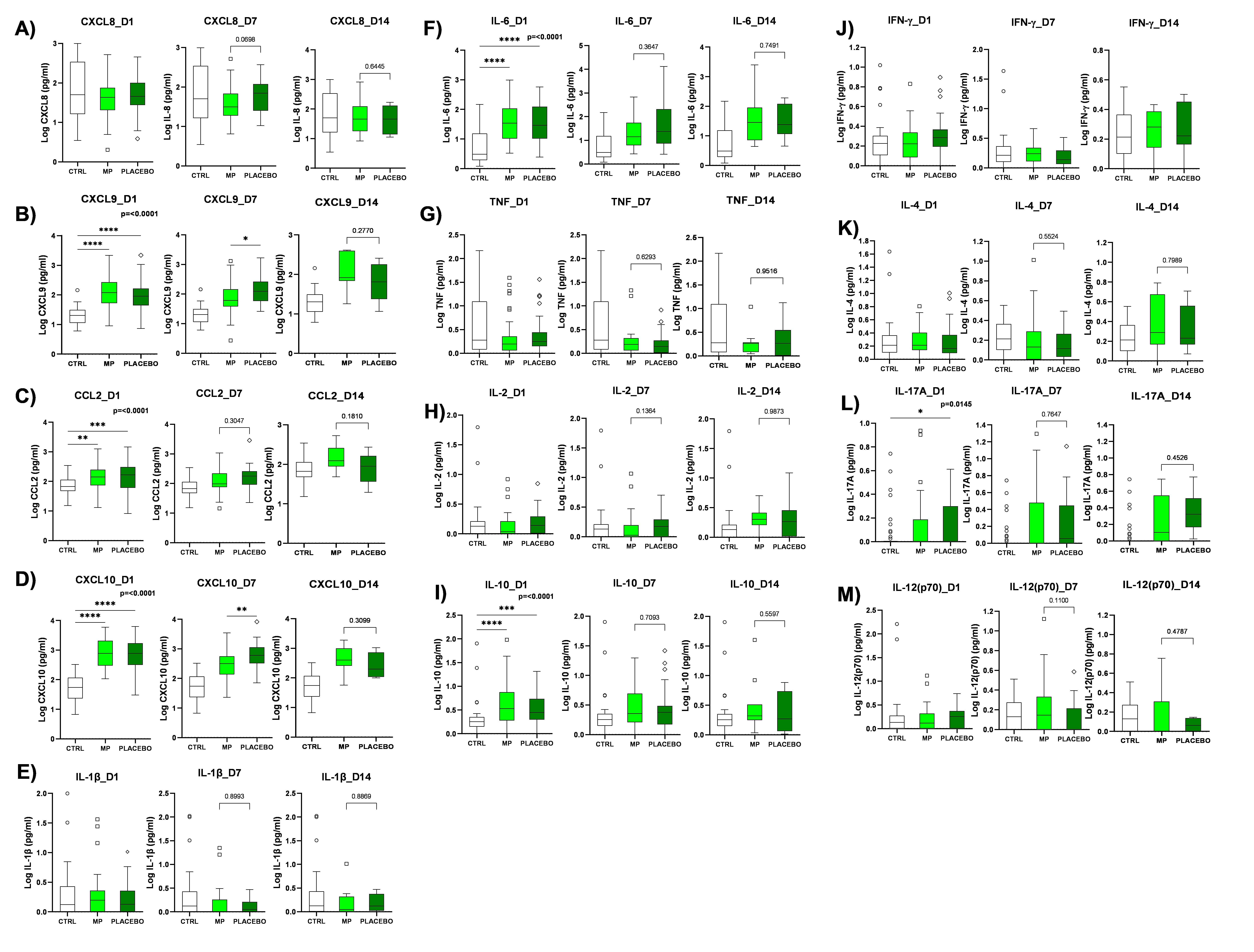

Supplement: Supplementary file 1 [file Image_1.jpeg]
